# Making a HIIT: study protocol for assessing the feasibility and effects of co-designing high-intensity interval training workouts with students and teachers

**DOI:** 10.1186/s12887-022-03440-w

**Published:** 2022-08-05

**Authors:** Stephanie L. Duncombe, Alan R. Barker, Lisa Price, Jacqueline L. Walker, Paul E. Dux, Amaya Fox, Natasha Matthews, Michalis Stylianou

**Affiliations:** 1 School of Human Movement and Nutrition Sciences, The University of Queensland, Saint Lucia, Queensland 4072 Australia; 2 Children’s Health and Exercise Research Centre, Sport and Health Sciences, College of Life and Environmental Sciences, University of Exeter, Exeter, UK; 3 School of Psychology, The University of Queensland, Saint Lucia, Queensland 4072 Australia

**Keywords:** Exercise, Educational settings, Cardiometabolic health, Program evaluation, Wellbeing

## Abstract

**Background:**

High-intensity interval training (HIIT) is an effective strategy for improving a variety of health outcomes within the school setting. However, there is limited research on the implementation of school-based HIIT interventions and the integration of HIIT within the Health and Physical Education (HPE) curriculum. The aims of the *Making a HIIT* study are to: 1) describe the methodology and evaluate the feasibility of co-designing HIIT workouts with students and teachers in HPE; 2) determine the effect of co-designed HIIT workouts on cardiorespiratory and muscular fitness, and executive function; 3) understand the effect of co-design on students’ motivation, enjoyment, and self-efficacy towards the workouts; and 4) evaluate the implementation of the intervention.

**Methods:**

Three schools will participate. Within each school, three different groups will be formed from Year 7 and 8 classes: 1) Co-Designers; 2) HIIT Only; and 3) Control. The study will include two phases. In phase one, Group 1 will co-design HIIT workouts as part of the HPE curriculum using an iterative process with the researcher, teacher, and students as collaborators. This process will be evaluated using student discussions, student surveys, and teacher interviews. In phase two, Groups 1 and 2 will use the co-designed 10-minute HIIT workouts in HPE for 8-weeks. Group 3 (control) will continue their regular HPE lessons. All students will participate in cardiorespiratory fitness, muscular fitness, and executive function assessments before and after the HIIT program or control period. Students will complete questionnaires on their motivation, enjoyment, and self-efficacy of the workouts. Differences between groups will be assessed using linear regressions to account for covariates. Heart rate and rating of perceived exertion will be collected during each HIIT session. The implementation will be evaluated using the Framework for Effective Implementation. Ethical approval was granted by the University of Queensland Human Research Ethics Committee and other relevant bodies.

**Discussion:**

This study will be the first to co-design HIIT workouts with teachers and students within the HPE curriculum. As this study relies on co-design, each HIIT workout will differ, which will add variability between HIIT workouts but increase the ecological validity of the study.

**Trial registration:**

ACTRN, *ACTRN12622000534785*, Registered 5 April 2022 – Retrospectively registered, https://www.anzctr.org.au/ACTRN12622000534785.aspx

**Supplementary Information:**

The online version contains supplementary material available at 10.1186/s12887-022-03440-w.

## Background

Physical inactivity is an important global issue as a high proportion of children and adolescents are not achieving the recommended levels of physical activity for health benefits [1, 2]. Evidence suggests that increasing vigorous physical activity is particularly important as it could be driving health benefits [3, 4]. High-intensity interval training (HIIT) is a method of acquiring vigorous physical activity and includes short bouts of high-intensity exercise interspersed with recovery periods [5]. HIIT is becoming a popular tool for physical activity acquisition in schools and HIIT interventions have been linked with improvements in markers of body size and composition, blood biomarkers, and cardiorespiratory fitness [6]. HIIT is also structured similarly to children’s intermittent patterns of physical activity [7] and can offer opportunities to facilitate learning in health and physical education (HPE) lessons [8].

Schools are an opportune environment to implement HIIT interventions as they can reach a large proportion of adolescents, have existing facilities, and staff capable of facilitating HIIT sessions [9]. Yet, research in schools can present several challenges, including the risk of overburdening teachers or taking away valuable time from the curriculum [9]. To date, most HIIT interventions have not adapted to these challenges and have been conducted during HPE lesson time with no links to the curriculum [6]. Further, very few HIIT interventions have incorporated student and teacher input into the workouts used and none have investigated designing the workouts within the curriculum [6]. Therefore, reviews focused on the topics of school-based HIIT and HIIT in children and adolescents have recommended consulting students and teachers on the design and evaluation of the intervention, and investigating the integration of HIIT within the curriculum [6, 10, 11].

Inherently, integrating HIIT into the curriculum requires the involvement of teachers and students. According to the International Association for Public Participation, engaging end-users in programs exists across a 5-stage continuum ranging from informing to empowering [12]. While stage 5 (empowering) enables the highest level of engagement, it is not always feasible in the curriculum due to time constraints and assessment requirements. However, lower levels of participation, such as involvement or collaboration, where end-users are involved in each phase of the process, are still viewed as beneficial. This active collaboration is often referred to as co-design, which is defined as collective creativity across the entire design process [13, 14]. In the current study, co-design presents a unique opportunity to combine the expertise and lived experiences of researchers, teachers, and students on: 1) the topic of HIIT; 2) the curriculum and school setting; and 3) their physical activity participation. Co-designing HIIT workouts within the curriculum has the potential to support educative outcomes and aligns with several Australian HPE curriculum content descriptions for Year 7 and 8 including, designing personal fitness plans, measuring heart rate, and predicting the benefits of physical activity for health [8, 15]. Further, collaboration with students and teachers in designing the workouts and intervention could increase the chances of implementation and engagement in the school setting as it is tailored to meet the needs and interests of end-users [13].

A limited number of studies have conducted process evaluations to assess the implementation of HIIT interventions in schools [16, 17]. Other studies have reported only selected aspects of process evaluations within their overall results, such as the dosage delivered and received, which could potentially lead to biased results [6, 11]. Process evaluations are important for understanding the connection between implementation and any null, negative, or positive findings [18]. For intervention studies employing co-design, evaluating both the process of co-design and the implementation of the intervention is necessary to draw appropriate conclusions.

### Aims and objectives

The *Making a HIIT* study aims to examine the process and effectiveness of co-designing and implementing HIIT workouts with secondary school students and teachers within HPE. *Making a HIIT* will be conducted in two phases with the following objectives in each phase:

#### Phase one:

1. To describe the methodology and results of the co-design process to develop HIIT workouts in the HPE curriculum, using the framework outlined by *Leask* et al. [19].
2. To evaluate the feasibility of co-designing HIIT workouts with students and teachers as part of the HPE curriculum.

#### Phase two:

1. Determine the effect of a HIIT intervention on cardiorespiratory fitness, muscular fitness, and executive function.
2. Determine the effect of the co-design process on students’ enjoyment, self-efficacy, affect, basic psychological needs, and motivation towards the HIIT workouts.
3. Evaluate whether the intervention was implemented as intended through a process evaluation using the framework described by *Durlak and DuPres* [20].

## Methods/design

### Overview

**Fig. 1:**
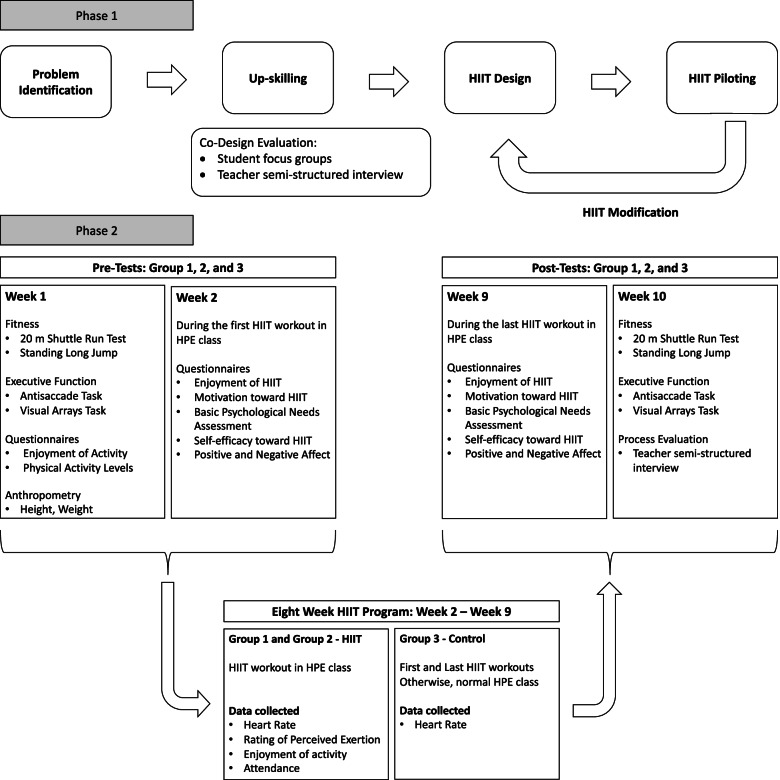
Overall Study Schematic. Overall study schematic outlining the lesson topics that will be used in phase one to co-design HIIT workouts with Group 1 and the intervention using the HIIT workouts in phase two. The pre-test and post-test measures are listed under their respective weeks. The data that will be collected during the intervention for the groups performing HIIT (Group 1 and 2) and for the control group (Group 3) are displayed under the eight-week HIIT program. HIIT = high intensity interval training; HPE = health and physical education

*Making a HIIT* will be completed in two phases, occurring during two subsequent terms in the same school year for each participating school. In phase one, students will co-design HIIT workouts with teachers and researchers as a part of their HPE curriculum (Fig. 1). The process will be evaluated using student discussions, student written feedback, and teacher interviews.

In phase two, the HIIT workouts designed in phase one will be implemented using an intervention with a quasi-experimental design. All consenting students will participate in cardiorespiratory fitness, muscular fitness, and executive function assessments before and after the HIIT intervention or control period. They will also complete questionnaires on their motivation, enjoyment, and self-efficacy towards HIIT as displayed in Fig. 1.

### Grounding theories

The theory of expanded, extended, and enhanced opportunities states that more physical activity can be accrued if students are provided with more opportunities, more time for these opportunities and higher quality opportunities for routine physical activity [21]. In line with this theory, *Making a HIIT* aims to enhance HPE lessons by introducing curriculum content targeting high-intensity physical activity and using co-design, which could potentially enhance student engagement throughout the intervention [21]. *Making a HIIT* also includes components that support the basic psychological needs (autonomy, competence, and relatedness) described within self-determination theory (SDT), including the co-design process, exercise modifications, and partner and group workouts [22, 23]. The combined use of these two theories to inform the study aim to support meaningful opportunities for physical activity and foster students’ motivation to participate.

### Recruitment and participants

Schools in greater Brisbane will be recruited to *Making a HIIT* through purposeful sampling. Schools with known contacts will be identified and contacted one by one until three schools agree to participate. The aim is to consecutively recruit one co-educational school, one boys’ school, and one girls’ school. The first school will be used as a pilot school to trial the co-design lessons and adapt them if needed before starting in the next two schools. It will include all of the phase one activities and the questionnaires pertaining to students’ motivation, enjoyment, and self-efficacy towards HIIT. The range of included schools aims to help understand potential sex and school-based differences to comprehensively evaluate the integration of co-designing HIIT within HPE.

The head of the HPE department and HPE teachers will be informed of the study and will be involved in class selection for the study. Interested teachers will need to provide informed consent to participate. All students in the classes of consenting teachers will be eligible to participate as *Making a HIIT* will be conducted as part of the curriculum. However, only students who provide parental consent and student assent will have their data collected as part of the study. Students will be excluded if they are unable to participate in the HIIT workouts due to injuries or other reasons. Students will be in either Year 7 or Year 8 (aged 12–14 years) as these 2 years share the same curriculum content descriptions [15]. The flexibility of using Year 7 or 8 allows schools to meaningfully integrate the work conducted as part of the study in their curriculum according to their local needs.

Within each school, *Making a HIIT* will recruit three groups of participants: Group 1) HPE classes involved in the co-design of the HIIT workouts in phase one and who use the HIIT workouts in phase two (Co-Designers); Group 2) HPE classes that use the HIIT workouts in phase two but are not involved in the co-design (HIIT only); and Group 3) HPE classes that continue normal HPE lessons in phase two and are not involved in the co-design (control group). In the pilot school, only one class will be recruited for each group. In the second and third school, two classes will be recruited for each group.

### Sample size calculation

The sample size calculation was based on the main outcome of phase two, which is cardiorespiratory fitness using the 20-m shuttle run test (20mSRT). An achievable and meaningful difference has previously been reported as between 5 and 6 laps in adolescents for HIIT-based interventions [24, 25]. Six laps will be used in this study. The standard deviation of the 20 m SRT test was 22 laps in a sample of 100 school children aged 13–15 years [24]. Using 3 groups, an α of 0.05, and a power of 80%, we anticipate needing 44 students in each of the three groups based on a G*Power (Version 3.1) calculation (Critical F = 3.066, df = 2) [26]. Based on expected recruitment rates (75–80%), typical class sizes (25–30 students), attrition (5%), and data loss due to absence or abstaining from specific measures (10–15%) [17, 24, 27], two classes will be recruited to each group in schools two and three.

### Phase one

Phase one will use a co-design process with researchers, teachers, and students. Only students in Group 1 will participate in this phase. The co-design will be conducted as part of the HPE curriculum. The Australian curriculum includes content descriptions related to designing fitness plans and modifying systems to allow students to enjoy and succeed [15], which are aligned with the co-design of HIIT workouts.

The development of the HIIT workouts will take place during obligatory HPE lessons. Students will complete approximately 6 lessons focused on problem identification, upskilling, design, and modification in an iterative process as recommended in the Framework by *Leask* et al. [19]. The number of lessons can be adjusted to meet the needs of participating schools but will encompass the topics outlined in Table 1. The pedagogical strategies will also be modifiable based on the needs of schools and teachers. The lessons may occur during different content units depending on the school and teacher. The same researcher will facilitate the lessons during the co-design process in each participating class.

**Table 1:** Topics covered in HIIT Co-design

**Problem Identification**	• Students brainstorm their barriers and facilitators to exercise • Students group their barriers and facilitators into main themes visually with sticky notes • Collectively, students, teachers, and researchers, use the barriers and facilitators to create evaluation criteria that can be used to design and evaluate HIIT workouts
**Upskilling**	• In groups, students discuss what they already know about HIIT • Students explore their heart rate using monitors and Polar GoFit software that shows intensity levels in different colours • Students partake in several HIIT workouts, reflect on their heart rate, and rate the workouts using their class criteria
**HIIT Design**	• Collectively decide, with students, teachers, and researchers, the percentage of heart rate maximum to be classified as high-intensity, and the minimum and maximum interval lengths prior to starting the workout design (HIIT parameters) • In groups, students identify potential themes for HIIT workouts and exercises that fit the theme • In groups, students create HIIT workouts that meet both the HIIT parameters and their evaluation criteria
**HIIT Piloting**	• Each group presents their workout to the class with the aid of the teacher and researchers • Students provide feedback on other groups’ HIIT workouts based on their evaluation criteria, and teachers and researchers to provide one or two comments • Heart rate for each pilot is recorded
**HIIT Modification**	• Each group modifies their workouts based on 1) their own reflection of their pilot; 2) feedback from the other students, researchers, and teachers; and 3) the heart rate summary data from their pilot

The co-design team will include the researcher, the teacher, and the students in each class. The aim and purpose of the sessions will be discussed by each co-design team and students and teachers will be able to provide feedback on the activities and pedagogical strategies used. Each member of the co-design team will be encouraged to share their experiences and expertise through activities designed to elicit collaboration. The co-design team will collectively define the parameters for the HIIT workouts (high intensity threshold and interval length) and criteria for an enjoyable HIIT workout. Based upon these parameters, students will co-design HIIT workouts in small groups, and will subsequently have the opportunity to trial their workouts and receive feedback from the co-design team based on the criteria established and heart rate data. To complete phase 1 activities, the student groups will have an opportunity to modify their workouts based on the feedback they receive.

At the end of the phase one, students will reflect on the co-design process through group discussions and individual written feedback to explore their thoughts about educative outcomes from the lessons, lesson aspects that were enjoyable or beneficial and in what way, their intentions to use the workouts in the future, and suggestions for any changes to the process used. Semi-structured interviews will be conducted with teachers to understand their thoughts on how the co-design lessons related to the curriculum and supported educative outcomes, their intentions to use co-design in the future, and their thoughts on student engagement during the co-design lessons.

### Data collected

Throughout phase 1, the following data will be collected to support the reporting of the methods and results of the co-design process: 1) the criteria created by students to evaluate the workouts; 2) students’ evaluations of pre-made HIIT workouts using their criteria; 3) students’ decisions on HIIT parameters (intensity and interval length); 4) draft HIIT workouts; 5) peer feedback using the criteria on the draft HIIT workouts; 6) heart rate data on draft HIIT workouts; and 7) finalised HIIT workouts.

To evaluate the co-design process, both student and teacher feedback will be collected throughout the process. After each lesson, students will have the opportunity to reflect on the activities and pedagogical strategies used and ask questions or provide suggestions and requests for future lessons, either verbally or written anonymously on an index card. At the end of the process, audio recordings of teacher interviews, notes from student discussions, and individual written feedback from students will be collected to examine the feasibility of co-designing HIIT workouts within the HPE curriculum and relevant outcomes.

### Data analysis

Descriptive statistics will be used to report the methods and results of the co-design process at each school. For example, they will be used to summarise student evaluations of the various HIIT workouts, highlight differences between schools in the created criteria, HIIT parameters, and HIIT workouts. To evaluate the co-design process, a thematic analysis will be completed using students’ feedback about the lesson, notes from student group discussions, individual student written feedback, and semi-structured teacher interviews at the end of the term. The thematic analysis will include familiarisation with the dataset, coding during a re-read of the dataset, and theme development [28, 29]. At least two authors will be involved in refining the themes. Participating teachers will be involved in member checking for feedback on the generated themes. Lastly, the themes will be presented beside quotes which exemplify each theme [28].

### Phase two

Phase two will use a quasi-experimental design and follows the SPIRIT checklist (Additional file 1).

### Intervention

The intervention will consist of the HIIT workouts designed by Group 1 in phase one. Decisions about the delivery, including the number of HIIT sessions per week and the types of lessons where HIIT is delivered (e.g., theory and/or practical lesson), will be made with teachers, and informed by the local school context. The aim is to have two workouts per week completed in practical and/or theory lessons for a duration of 8 weeks within a single 10-week term. Each HIIT workout will take approximately 10-minutes to complete and will be led by the HPE teacher of each class. For teachers who were not involved in the co-design process, a meeting will be scheduled to discuss the HIIT workouts and expectations for the intervention. All involved teachers will also receive a booklet with the HIIT workout (intervals, exercises with descriptions and modifications, and necessary equipment) for each week. The intervention will be completed by Groups 1 (Co-Designers) and 2 (HIIT only). Following the HIIT workout, HPE lessons will continue as normal. Group 3 (Control) will complete the first and last HIIT workout in the 8-week intervention to be able to appropriately respond to the questionnaires focused on HIIT but will otherwise continue with normal HPE lessons.

### Pre-intervention and post-intervention measurements

All intervention measures will be collected by research assistants blinded to group allocation. Baseline measurements will include anthropometry, general enjoyment of physical activity, and self-reported physical activity levels (Fig. 1). These measurements will take place the week prior to the intervention and require approximately 15 minutes to complete. Measurements for cardiorespiratory fitness, muscular power, and executive function will be conducted 1 week prior to and 1 week post intervention and require 60 minutes to complete. Questionnaire data relating to motivation, basic psychological needs, enjoyment, positive and negative affect, and self-efficacy will be collected after the first and last HIIT workout for all groups and will be completed in 10 minutes. Data will be collected using the same protocols at both timepoints. These data will be collected in all 3 groups during HPE lessons.

#### Anthropometry

Students will be asked to remove shoes, hats, and any heavy or bulky clothing. Stature will be measured using a stadiometer. Students will be asked to stand with their feet together and have their heels against the back of the stadiometer while keeping their knees straight. They will be instructed to breathe in and stand tall. Their stature will be recorded to the nearest 0.01 m. Body mass will be measured using a calibrated scale. Students will be asked to stand on the scale facing forward with their arms by their side. Their body weight will be recorded to the nearest 0.1 kg. Body mass index (BMI) will be calculated as (body mass (kg) divided by stature (m) squared). To determine students’ weight categories, age and sex specific BMI cut points will be used [30]. Based on discussions with key stakeholders (school gatekeepers and teachers), these measures will only be collected at the baseline visit and will be used as covariates in the study to understand differences based on body weight status.

#### General physical activity levels

Baseline general physical activity level data will be collected using the physical activity questionnaire for children (PAQ-C), a self-administered, 7-day recall instrument that generates a physical activity score based on eight items scored on a 5-point scale [31]. The questionnaire is reliable (ICC = 0.96) [32], and its convergent validity is supported through relationships with an activity rating question, a teacher’s rating of physical activity, and moderate to vigorous physical activity assessed by a separate inventory (*r* = 0.45 to *r* = 0.63) [33, 34]. Further, the PAQ-C is quick to complete (approximately 5 minutes), [35] and is one of three physical activity measures that received majority support within an expert group [34]. This questionnaire will only be completed prior to the intervention to understand participants baseline general physical activity levels, which will be used as a covariate in data analysis.

#### Cardiorespiratory fitness

The 20 m SRT will be used to measure cardiorespiratory fitness [36]. The test involves continuous running between two lines in time to recorded beeps with speed that increases by 0.5 km/hr at each level. It requires minimal equipment and can be administered to a large number of students simultaneously [37]. It is easy to administer and is time efficient, with a maximum test option lasting 22 minutes [37]. Additionally, it is a test that students typically engage with during HPE as part of the curriculum. The 20 m SRT is the most used field test for cardiorespiratory fitness [38]. It has a moderate to high criterion-related validity against peak oxygen uptake (*r*_p_ = 0.62–0.84) in adolescents according to both a relevant meta-analysis [39] and systematic review [38]. The number of laps each participant completes will be recorded. The 20 m SRT will be used as an outcome variable examining differences between groups over time.

#### Muscular fitness

The standing long jump will be used to measure muscular power. This jump involves a two-foot take-off and landing. Students will stand behind a line with both feet and will be encouraged to bend their legs and swing their arms for maximum forward movement. The distance of the jump will be recorded from the line to the back of the student’s foot. Each student will have three attempts. The standing long jump is a practical, time-efficient, and low-cost test [40, 41]. It is valid (*r* = 0.70 with 1 repetition leg extension) and strongly associated with other lower body strength tests (*r* = 0.83–0.86) and upper body strength tests (*r* = 0.69–0.85), making it a general indicator of muscular fitness in youth [40–42]. It is commonly used within the HPE curriculum [25, 43–46]. The standing long jump will be used as an outcome variable examining differences in groups and time.

#### Executive function

An antisaccade task and a visual array task will be conducted on computers using PsychoPy software [47]. These tasks will be used to assess students’ selective attention, inhibition, and working memory and will take approximately 30 minutes to complete [48]. The tasks were pilot tested with Year 8 students and modified appropriately.

The antisaccade task measures inhibitory control of attention and has previously been used in an exercise intervention trial with adolescents [49]. The task will be conducted as previously described [48]. In brief, students will focus on a fixation cross in the centre of their screens. After a visual cue, an asterisk will appear on one side of the screen, followed by a Q or O on the opposite side that is immediately covered by “##”. Students will be told to ignore the asterisk and respond to the Q or O. Due to the classroom setting, the original audio cue for this task was replaced with a visual cue to minimise distraction to other students. Prior to starting the task, students will receive a practice round. They will receive feedback throughout the task on their answers and will be provided with a break in the middle of the 72 responses. This task has good internal consistency (*r* = 0.92) and test-retest reliability (*r* = 0.71) [48]. The number of correctly identified target letters will be used as the outcome variable from the antisaccade task to examine differences between groups over time.

The visual arrays task, which provides a measure of the capacity of working memory and selective attention ability, will also be completed as previously described [48]. Students will see an array of blue and red rectangles flash on screen after being told to focus on one of the two colours. Subsequently, only the colour they were told to remember will appear on screen with one rectangle labelled using a white dot. Students will be asked if that rectangle has changed orientation from the original display. During pilot testing, each array contained five or seven rectangles of each colour. Based on the results and student feedback, an array with three rectangles of each colour was included to ensure an appropriate dosage curve. Before the task begins, students will have two practice rounds. This includes one round with a longer initial flash and one round at full speed. Students will receive feedback throughout the practice, but not during the actual task. The visual arrays task has good internal consistency (*r* = 0.75) and test-retest reliability (*r* = 0.67) [48]. It has previously been used in children as young as 10 years old [50]. The outcome variable of interest from the visual array task is the capacity score *(k)*, which provides a measure of working memory capacity. It is calculated from *N* × (Hits + Correction + Rejections − 1), where *N* is the set-size for that array [48].

#### Motivation

Motivation towards HIIT will be measured using the perceived locus of causality (PLOC) questionnaire that was developed by Goudas et al [51] based on the original questionnaire by Ryan and Connell [52]. It has been used extensively to assess motivation in HPE [53]. The questions are administered using a 7-point Likert scale. For this study, we changed the stem from “I take part in PE/sport” to “I take part in HIIT workouts …” . The questions are based on SDT and assess motivation, external regulation, introjected regulation, identified regulation, and intrinsic regulation to gain an understanding of what motivates students to participate in HIIT [51]. The PLOC will be used as an outcome variable examining differences in groups and time.

#### Basic psychological needs

Three innate psychological needs are encompassed within SDT: autonomy (the need to be self-governed), relatedness (need to feel connected and accepted by others), and competence (the need to succeed in various tasks) [22]. The basic needs theory within SDT hypothesizes that when these needs are met students will have improved intrinsic motivation, wellbeing, and health [22, 54, 55]. Five 7-point Likert scale questions will be used to assess each need. Autonomy during the HIIT workout will be assessed using the questions collated by Standage et al. (2003), who demonstrated internal reliability of the questions in an HPE setting [56]. Relatedness during the HIIT workout will be assessed using a subscale of the Need for Relatedness Scale [57], which has previously been used in HPE with acceptable internal reliability [56, 58]. Lastly, HIIT competence will be assessed using 5 questions from the perceived competence subscale of the intrinsic motivation inventory [59], which has also been shown to be reliable in an HPE setting [56, 60]. The basic psychological needs will be used as an outcome variable examining differences in groups and time.

#### Enjoyment

Enjoyment of general physical activity will be measured for all groups using physical activity enjoyment scale (PACES) before the intervention [61]. The version used in adolescents and youth includes the prompt “When I am active” and 16 phrases that students rank on a 5-point Likert scale from 1 (Disagree a lot) to 5 (Agree a lot) [62]. The overall score is calculated by summing the 16 responses and dividing by the number of questions, with a higher score demonstrating greater enjoyment. It has been validated for both children and adolescents [63]. Enjoyment of general physical activity will be used as a covariate for understanding enjoyment of HIIT specifically.

Enjoyment of HIIT will be also measured using the PACES questionnaire. The stem of the questionnaire will be changed from “When I am active” to “When I am participating in HIIT”. Enjoyment has previously been shown to mediate the effects of school-based physical activity interventions and will be a key variable to examine in this study [64]. The PACES questionnaire on HIIT will be used as an outcome variable examining differences in groups and time.

#### Positive and negative affect

A 9-item questionnaire will be used to assess affect using the prompt “During this workout, I felt”. Students will respond on a 5-point Likert scale from 1 (Not at all) to 5 (Extremely). Five items on the scale are related to positive affect (e.g., proud) and four to negative affect (e.g., unhappy). This is a short-form of the original positive and negative affect scale (PANAS) that was developed to be used in children and has shown to be reliable [65]. This questionnaire will be used as an outcome variable examining differences in groups and time.

#### Self-efficacy

Self-efficacy toward HIIT will be measured using the HIIT-SEQ, which has previously been used with and validated for adolescents [66]. It includes 6 questions on a 10-point Likert scale. It will be an outcome variable used to understand differences in students’ confidence in relation to performing HIIT workouts between groups and over time.

### Intervention measurements

#### Heart rate

Heart rate will be monitored throughout the HIIT sessions and the remainder of the HPE lessons to evaluate intensity for Groups 1 and 2 using Polar H10 monitors (Polar H10, Polar Electro, Finland). Heart rate will also be monitored throughout the HPE lesson in Group 3. Polar H10 monitors will be provided to students and the Polar GoFit system (https://polargofit.com/) will be used to collect data. This will be done anonymously using a number assigned to each student. Students’ maximum heart rates will be determined during the baseline 20 m SRT. Heart rate will be used to assess the fidelity of the HIIT workouts through the calculation of average and peak heart rate, the percentage of students above the thresholds of 80 and 90% of maximum heart rate, and the percentage of time students spend in various deciles (> 80% maximum heart rate, > 90% maximum heart rate).

#### Rating of perceived exertion

The omnibus (OMNI) children’s rating of perceived exertion (RPE) scale will be administered at the end of each HIIT workout for Groups 1 and 2 and at the end of the first and last HIIT workout for Group 3. Students will reflect on how tired they were throughout the entire HIIT session using the prompt “During this workout, I felt”. They will respond using a pictorial scale from 0 (not tired at all) to 10 (very, very tired) [67]. The OMNI-RPE scale has demonstrated strong criterion validity for walking/running against both heart rate and peak oxygen consumption in children aged 11–12 and ≥ 13 years (*r* ≥ 0.82) [68]. A sessional score will be calculated by multiplying the RPE by the duration of the session to represent the load for the entire session. The sessional RPE will be used to assess the fidelity of the HIIT workouts.

#### Enjoyment of HIIT workout

One 5-point Likert scale question will be used to assess students’ enjoyment at the end of each HIIT workout for Groups 1 and 2. Students will rate their enjoyment using the following prompt: “I enjoyed participating in today’s HIIT session” between 1 (strongly disagree) and 5 (strongly agree). A similar style question has previously been used to assess student satisfaction of HIIT workouts [45]. This will be used to understand changes over time in enjoyment and enjoyment of specific workouts and student responsiveness.

#### Process outcomes

The process evaluation will be guided by the Framework for Effective Implementation [20]. The number of schools and students contacted about the study will be tallied to inform the recruitment rate. The number of HIIT workouts delivered by teachers and attended by students will be recorded to inform the dosage delivered and received. Heart rate and RPE will be recorded to assess fidelity to high intensity. Heart rate will also be used to monitor intensity during regular HPE lessons in the control group. Enjoyment of each workout will be recorded to understand student responsiveness. Any adverse events will be recorded by a researcher in a logbook. Any modifications made to the workouts will be recorded by a researcher. Semi-structured interviews will be completed with teachers at the end of phase two on the topic of implementation to investigate adaption and quality.

### Data analysis

Data entry will be completed by one researcher with at least 10% checked by a second researcher. All data will be checked prior to analysis using range to assess any outliers or errors in data entry. To describe the population, descriptive statistics will be reported for each school separately. To determine the effect of the HIIT intervention on cardiorespiratory fitness, muscular fitness, and executive function, general linear models will be used to assess changes in the dependent variables with group (HIIT or control), timepoint, and group x time interaction included as independent variables. To determine the effect of involvement in co-design on students’ motivation, enjoyment, self-efficacy, feelings, and basic psychological needs, generalised linear models will be used to assess changes in the dependent variable, with group (Co-Design or not), timepoint, and group x time interaction included as dependent variables. Potential covariates, such as sex, age, BMI, involvement in co-design, baseline physical activity levels, baseline physical activity enjoyment, and baseline levels of the dependent variable will be identified using two-by-two tests and included in the model where appropriate.

The implementation of *Making a HIIT* will be evaluated using the Framework for Effective Implementation [20] across 8 components: 1) program reach – number of consenting schools and students; 2) dosage – number of HIIT workouts delivered and completed; 3) fidelity – students’ heart rate and RPE during HIIT workouts; 4) quality – variation in heart rate between students; 5) monitoring of control group – via heart rate; 6) responsiveness – student enjoyment and teacher perspectives; 7) adaption – modifications of the HIIT workouts by teachers; 8) differentiation – uniqueness of study.

### Ethical considerations and dissemination

*Making a HIIT* has been approved by The University of Queensland’s human research ethics committee (Project: 2020/HE002444) and school organisations as necessary. All researchers involved in the study will have to complete appropriate checks and training to ensure child safety. Consent for participating schools will be provided by school gatekeepers (e.g., principals). Involved teachers will also provide consent. All students will partake in designing and participating in the HIIT workouts as it will be completed as part of the curriculum. However, only students who have both parental/guardian consent and student assent will have their data collected as part of this study. All data collected will be stored anonymously on a secure server. Results of *Making a HIIT* will be disseminated through publications in peer-reviewed journals and conference presentations.

## Discussion

This paper presents the protocol for the *Making a HIIT* study. *Making a HIIT* will include the novel component of co-designing HIIT workouts with students and teachers within the HPE curriculum. Further, it will examine if co-designing HIIT workouts affects student engagement during the workouts and if it moderates any outcome variables. *Making a HIIT* will include three different school types (co-educational school, boys’ school, and girls’ school) to provide insight into the integration of this type of work within a variety of schools and HPE units to support the HPE curriculum and to investigate potential gender differences. Currently, there are limited process evaluations on HIIT interventions in schools [6]. *Making a HIIT* will aim to comprehensively evaluate the extent to which the intervention was completed as intended. This is pertinent to understanding the link between implementation and outcomes, especially given evidence supporting that high-intensity exercise could be driving health benefits. This study does not include randomisation as it is not feasible in our protocol based on school timetables and preferences for class involvement. However, quasi-experimental designs are widely used in school-based research and are useful for comparing groups and measuring change when randomisation is not possible [69]. Overall, the results of this study will provide useful insights into the meaningful implementation of school-based HIIT interventions that support both educative and health outcomes.

## Supplementary Information

**Additional file 1.** SPIRIT Checklist, Checklist for Intervention Trials.

## Data Availability

Not applicable as this is a protocol paper so does not report on data.

## Abbreviations

BMI: Body mass index
HIIT: High intensity interval training
HPE: Health and physical education
IAP2: International Association for Public Participation
PACES: Physical activity enjoyment scale
PANAS: Positive and negative affect scale
PAQ-C: Physical activity questionnaire for children
PE: Physical education
PLOC: Perceived locus of control
OMNI: Omnibus
RPE: Rating of perceived exertion
SDT: Self-determination theory
SRT: Shuttle run test
